# Cyp19a1a Promotes Ovarian Maturation through Regulating E2 Synthesis with *Estrogen Receptor 2a* in *Pampus argenteus* (Euphrasen, 1788)

**DOI:** 10.3390/ijms25031583

**Published:** 2024-01-27

**Authors:** Chunyang Guo, Kai Zhang, Chang Li, Ruixue Xing, Shanliang Xu, Danli Wang, Xubo Wang

**Affiliations:** 1 College of Marine Science, Ningbo University, Ningbo 315211, China; guochunyang@nbu.edu.cn (C.G.); 206000792@nbu.edu.cn (K.Z.); lc18658152708@163.com (C.L.); 15990222981@163.com (R.X.); xushanliang@nbu.edu.cn (S.X.); wangdanli@nbu.edu.cn (D.W.); 2 Collaborative Innovation Center for Zhejiang Marine High-Efficiency and Healthy Aquaculture, Ningbo University, Ningbo 315211, China

**Keywords:** *Pampus argenteus*, aromatase, hormone, Cyp19a1a, *estrogen receptor*

## Abstract

In the artificial breeding of *Pampus argenteus* (Euphrasen, 1788), female fish spawn before male release sperm, which indicates rapid ovarian development. In fish, aromatase is responsible for converting androgens into estrogens and estrogen plays a crucial role in ovarian development. In this study, we aimed to investigate the potential role of brain-type and ovarian-type aromatase to study the rapid ovarian development mechanism. The results showed that *cyp19a1a* was mainly expressed in the ovary and could be classified as the ovarian type, whereas *cyp19a1b* could be considered as the brain type for its expression was mainly in the brain. During ovarian development, the expression of *cyp19a1a* in the ovary significantly increased from stage IV to stage V and Cyp19a1a signals were present in the follicle cells, while *cyp19a1b* expression in the pituitary gland decreased from stage IV to stage V. To further investigate the function of Cyp19a1a, recombinant Cyp19a1a (rCyp19a1a) was produced and specific anti-Cyp19a1a antiserum was obtained. The expressions of *cyp19a1a*, *estrogen receptors 2 alpha* (*esr2a*), and *androgen receptor alpha* (*arα*) were significantly upregulated in the presence of rCyp19a1a. Meanwhile, *cyp19a1a* was expressed significantly after E2 treatment in both ovarian and testicular tissue culture. Taken together, we found two forms of aromatase in silver pomfret. The ovarian-type aromatase might play an important role in ovarian differentiation and maturation, and participate in E2 synthesis through co-regulation with *esr2a.* The brain-type aromatase *cyp19a1b* might be involved in the regulation of both brain and gonadal development.

## 1. Introduction

*Pampus argenteus* is widely distributed in the Indian Ocean, North Sea, Oman Sea, and Persian Gulf, as well as from the Bohai Sea to the South China Sea in China [1,2,3]. *P. argenteus* is a commercially important fish in the aquaculture industry worldwide, and its artificial breeding technique has been studied in many countries [4,5]. With the breakthrough of *P. argenteus* aquaculture technology, artificial cultivation is gradually moving towards large-scale farming. However, in the farming production process, challenges such as high mortality rates, poor development, differences in meat quality, and disparities compared to wild *P. argenteus* still exist. The cultivation of *P. argenteus* also presents the phenomenon of females spawning before male fish release sperm, which increases the difficulty of fry rearing. In addition, the body size of female-cultured *P. argenteus* is larger than that of males at harvest, which reveals that it is important to study the gonad differentiation and development mechanism in artificial breeding of this species. The results will provide a theoretical basis for addressing related farming challenges of artificial cultivation and the establishment of mono-sex female production technology in *P. argenteus*.

Cytochrome P450 aromatase is a key steroidogenic enzyme that catalyzes the conversion of androgens to estrogens [6,7]. Two homologous genes encoding aromatase have been identified in fish, which are *cyp19a1a* and *cyp19a1b*. The *cyp19a1a* gene is primarily expressed in the gonads, whereas *cyp19a1b* is expressed in the brain [8]. Cyp19a1a, as a key enzyme for estradiol synthesis in teleosts, is crucial for gonadal differentiation, maintenance, and maturation [9]. Previous studies have shown that rapid downregulation of the *cyp19a1a* gene results in a significant decrease in the estradiol content in the serum of fish [10,11,12], and the gonads of protandrous hermaphroditic eels with a *cyp19a1a* knockout remain undifferentiated and do not develop into ovaries [13]. Meanwhile, Cyp19a1a is highly expressed in the yolk formation process [14,15,16], and has been proved to be involved in ovarian differentiation and vitellogenesis of oocytes in teleosts. In the pituitary gland of fish, *cyp19a1b* not only regulates the synthesis of estrogen through estrogen receptors but also controls the synthesis and secretion of follicle-stimulating hormone and luteinizing hormone by secretory cells, thereby regulating the differentiation, development, maturation, and spawning of gonads [17,18,19,20]. Both brain-type and ovarian-type aromatase play a regulatory role in the gonadal development of fish. However, there was no research on *P. argenteus*. Thus, this study aimed to study the ovarian development mechanism to explore the reason for rapid ovarian development in the artificial breeding of *P. argenteus* through investigating *cyp19a1a* and *cyp19a1b* gene expression patterns and potential functions during the reproductive cycle.

## 2. Results

### 2.1. Molecular Identification and Phylogenetics of the P. argenteus cyp19a1a and cyp19a1b Genes

**Figure 1:**
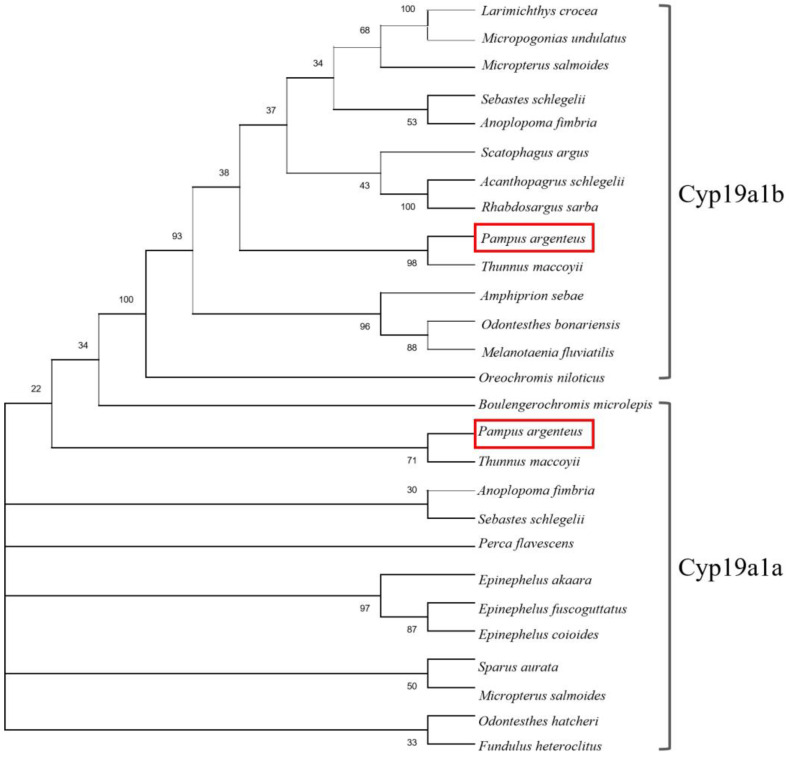
Comparison of the *P. argenteus* Cyp19a1a and Cyp19a1b protein sequences in different fish. The evolutionary distances were computed using the Jones–Taylor–Thornton (JTT) matrix-based method and are in the units of the number of amino acid substitutions per site. The rate variation among sites was modeled with a gamma distribution (shape parameter = 0.7). The confidence in the resulting tree was assessed through the bootstrap method with 1000 replications. The phylogenetic analysis of Cyp19a1a and Cyp19a1b was aligned using Clustal W. The GenBank accession numbers for sequence data analyzed with Cyp19a1a are as follows: *Anoplopoma fimbria* (AGH69789.1), *Fundulus heteroclitus* (AAR97268.1), *Odontesthes hatcheri* (ABK41198.1), *Sebastes schlegelii* (ACN39247.2), *Epinephelus fuscoguttatus* (AYQ58300.1), *Epinephelus coioides* (AAR97601.1), *Epinephelus akaara* (AY547354.1), *Perca flavescens* (ABL64075.1), *Sparus aurata* (AAL27699.1), *Boulengerochromis microlepis* (AGS14768.1), *Micropterus salmoides* (XP_038558015.1), *Thunnus maccoyii* (XP_042264966.1); and for Cyp19a1b: *Micropogonias undulatus* (AEL31294.1), *Larimichthys crocea* (ACO35042.1), *Sebastes schlegelii* (AGG53952.1), *Rhabdosargus sarba* (ABC70868.1), *Acanthopagrus schlegelii* (QEN95916.1), *Scatophagus argus* (AFV47578.2), *Amphiprion sebae* (AJP70568.1), *Melanotaenia fluviatilis* (AED99847.1), *Odontesthes bonariensis* (AAQ88434.2), *Oreochromis niloticus* (AAG18458.1), *Micropterus salmoides* (XP_038562227.1), *Thunnus maccoyii* (XP_042267530.1), *Anoplopoma fimbria* (XP_054476115.1). The two red frames indicated Cyp19a1b and Cyp19a1a in *P. argenteus*, respectively.

The sequences of the *cyp19a1a* and *cyp19a1b* genes were determined via ovary and brain cDNA cloning, respectively. The sequence of *cyp19a1a* (1536 bp) contained an open reading frame encoding 521 amino acids, and the sequence of *cyp19a1b* (1139 bp) contained an open reading frame encoding 379 amino acids. The phylogenetic analysis showed that Cyp19a1a and Cyp19a1b in *P. argenteus* were clustered with their homologous genes in other fishes (Figure 1).

### 2.2. Expression Profiles of cyp19a1a and cyp19a1b in P. argenteus

**Figure 2:**
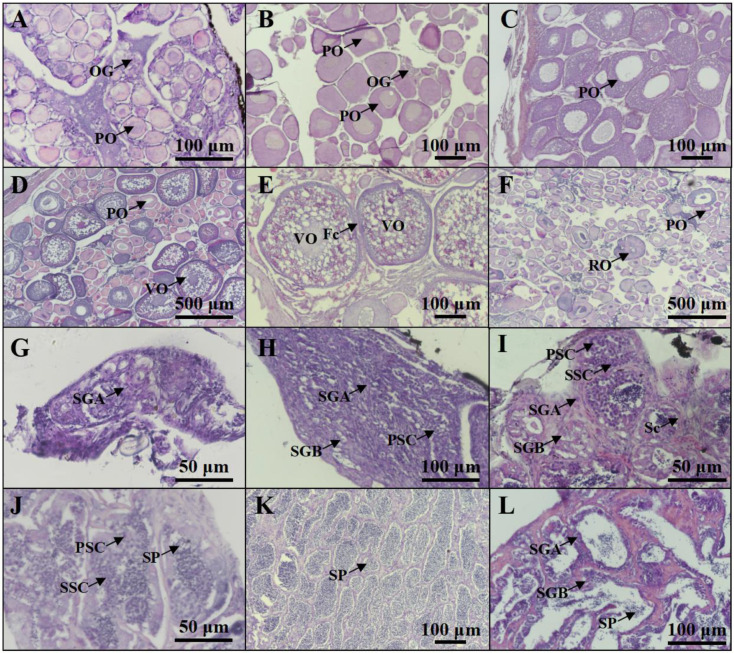
Profiles of gonadal development in *P. argenteus*. The different stages of ovarian development include stage I (**A**), stage II (**B**), stage III (**C**), stage IV (**D**), stage V (**E**), and stage VI (**F**). The different stages of testicular development include stage I (**G**), stage II (**H**), stage III (**I**), stage IV (**J**), stage V (**K**), and stage VI (**L**). OG, oogonia; PO, primary oocyte; VO, vitellogenic oocyte; Fc, follicle cells; RO, regressed oocyte; SGA, spermatogonia type A; SGB, spermatogonia type B; PSC, primary spermatocyte; SSC, secondary spermatocyte; Sc, Sertoli cell; SP, sperm.

**Figure 3:**
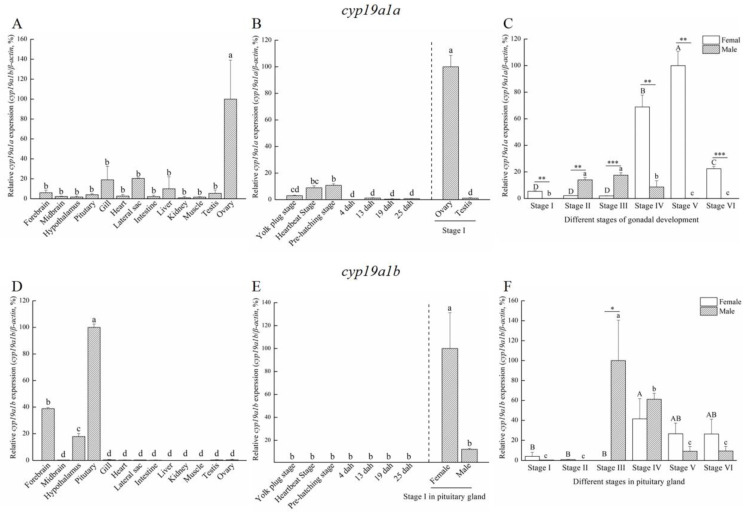
The expression patterns of *cyp19a1a* and *cyp19a1b* in *P. argenteus*. (**A**) The tissue distribution of *cyp19a1a* expression. (**B**) *cyp19a1a* expression during the early development stage. (**C**) *cyp19a1a* expression during gonadal development. (**D**) The tissue distribution of *cyp19a1b* expression. (**E**) *cyp19a1b* expression during the early development stage. (**F**) *cyp19a1b* expression during gonadal development. Different uppercase letters indicate one-way ANOVA, followed by Duncan’s test (*p* < 0.05) in female group. Different lowercase letters indicate one-way ANOVA, followed by Duncan’s test (*p* < 0.05) in male group. Asterisk indicates Student’s *t*-test (*p* < 0.05). “*” indicates Student’s t-test (*p* < 0.05). “**” indicates Student’s *t*-test (*p* < 0.01). “***” indicates Student’s *t*-test (*p* < 0.01).

Based on the histological characteristics, six different stages were classified in ovary and testis, respectively (Figure 2). The expression profiles of *cyp19a1a* and *cyp19a1b* were detected via qPCR. The qPCR results showed that *cyp19a1a* was significantly expressed in the ovary (Figure 3A), whereas *cyp19a1b* was significantly expressed in the pituitary, forebrain, and hypothalamus (Figure 3D). In the early development stage of *P. argenteus*, *cyp19a1a* was highly expressed from the yolk plug stage to the pre-hatching stage (Figure 3B). However, no significant change was found in *cyp19a1b* expression during the early development stage (Figure 3E). During gonadal development, *cyp19a1a* expression in ovaries was significantly higher than in testis (Figure 3C) with the highest expression at stage V in ovaries. The expression of *cyp19a1b* was significantly higher in males than in females at stage III (Figure 3F). In the pituitary gland, *cyp19a1b* expression decreased from stage III to stage V in males and stage IV to stage V in females.

### 2.3. Western Blot Analysis and the Localization of Cyp19a1a

**Figure 4:**
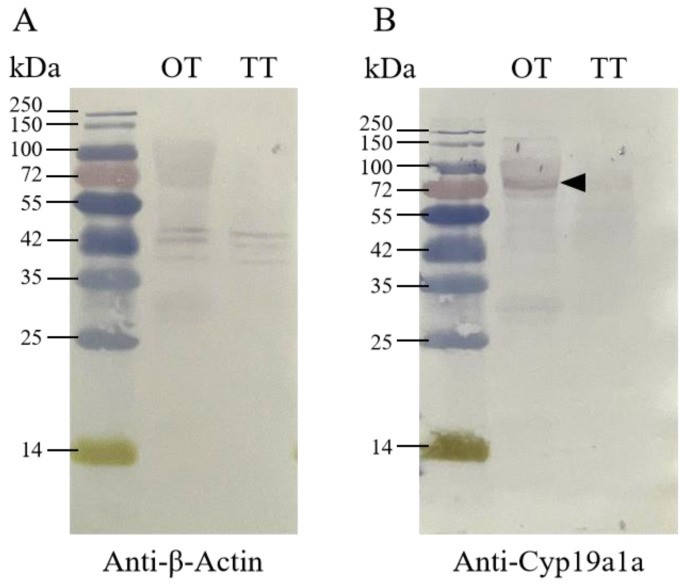
The Cyp19a1a protein in the gonad of *P. argenteus*. (**A**) The β-Actin signals were detected via anti-β-Actin antibody in ovarian and testicular tissue. (**B**) Cyp19a1a signals were detected via anti-Cyp19a1a antibody in ovarian and testicular tissue. The black arrowhead indicates the signal of the anti-Cyp19a1a antibody. OT, ovarian tissue; TT, testicular tissue. The black arrowheads indicate the signals of the anti-Cyp19a1a antibody.

**Figure 5:**
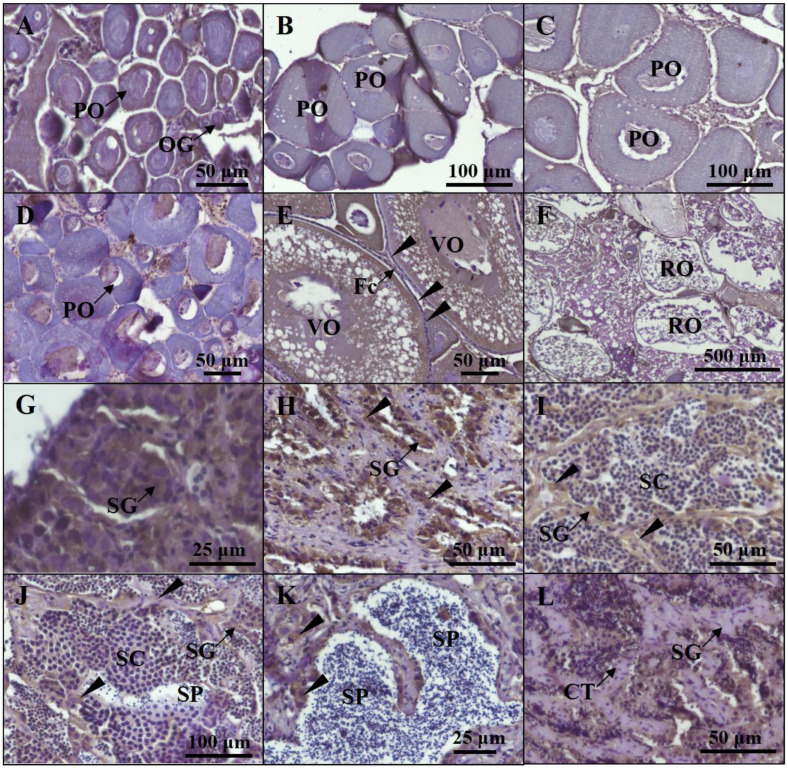
The distribution of Cyp19a1a protein at different gonadal stages. The distribution of the Cyp19a1a protein was detected via IHC staining at different gonadal stages, including stage I (**A**), stage II (**B**), stage III (**C**), stage IV (**D**), stage V (**E**), and stage VI (**F**) in females and stage I (**G**), stage II (**H**), stage III (**I**), stage IV (**J**), stage V (**K**), and stage VI (**L**) in males. OG, oogonia; PO, primary oocyte; Fc, follicle cell; VO, vitellogenic oocyte; RO, regressed oocyte; SG, spermatogonia; SC, spermatocyte; SP, sperm; CT, connected tissue. The black arrowheads indicate the positive signals of the anti-Cyp19a1a antibody.

For the Western blot analysis, the signal was observed in ovarian and testicular tissues using the anti-β-Actin antibody. According to Figure 4, β-Actin in *P. argenteus* was about 42 kDa, and Cyp19a1a at 75 kDa was detected using the anti-Cyp19a1a antibody in ovarian tissue. IHC was then used to detect Cyp19a1a protein expression at different gonadal development stages (Figure 5). The results showed that the signal of Cyp19a1a was only present in the follicle cells at stage V in the ovary (Figure 5A–F). In the testis, the signals were detected in spermatogonia from stage II to stage IV, which was consistent with the qPCR results (Figure 5G–L).

### 2.4. The Production of rCyp19a1a and the Role of rCyp19a1a in Gonadal Tissue Culture

**Figure 6:**
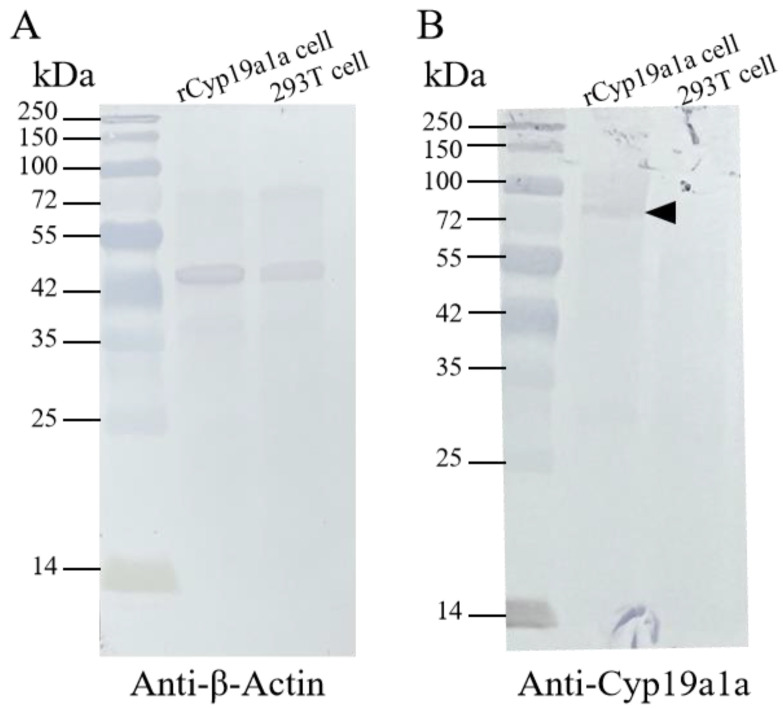
Western blot of recombinant Cyp19a1a produced in HEK293 cells. (**A**) The β-Actin signals were detected via anti-β-Actin antibody in rCyp19a1a cells and 293T cells. (**B**) The rCyp19a1a signals were detected via anti-Cyp19a1a in the rCyp19a1a cells and 293T cells. The black arrowhead indicates the signals of the anti-Cyp19a1a antibody.

**Figure 7:**
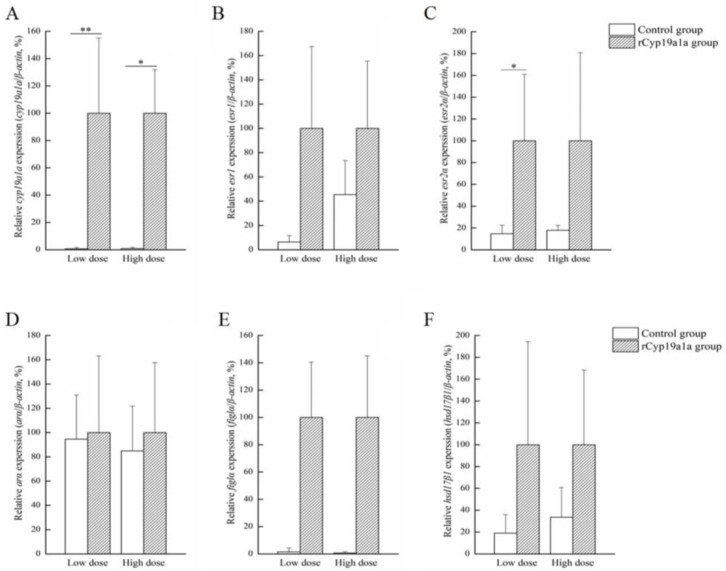
Effects of rCyp19a1a on sex-related gene expression in ovarian tissue. The expression of *cyp19a1a* (**A**), *esr1* (**B**), *esr2a* (**C**), *arα* (**D**), *figla* (**E**), and *hsd17β1* (**F**) in cultured ovarian tissues. The expression of sex-related genes was analyzed using qPCR (*n* = 6 for each value). “*” indicates Student’s *t*-test (*p* < 0.05). “**” indicates Student’s t-test (*p* < 0.01).

**Figure 8:**
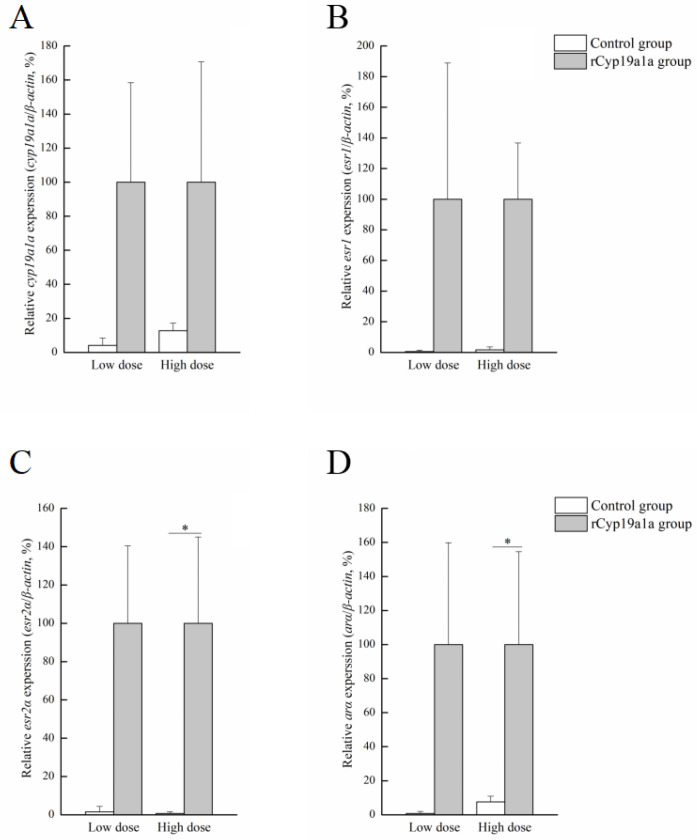
Effects of rCyp19a1a on sex-related gene expression in testicular tissue. The expression of *cyp19a1a* (**A**), *esr1* (**B**), *esr2a* (**C**), and *arα* (**D**) in cultured testicular tissues. The expression of sex-related genes was analyzed using qPCR (*n* = 6 for each value). “*” indicates Student’s *t*-test (*p* < 0.05).

rCyp19a1a was used to elucidate the function of Cyp19a1a through the in vitro ovarian and testicular tissue culture experiments. According to Western blotting, the Cyp19a1a signal was detected in rCyp19a1a cells, which was about 75 kDa (Figure 6). To further understand the role of Cyp19a1a in the gonads of *P. argenteus*, the in vitro system was used to study its effect on sex-related gene expression. The BrdU-incorporated cells were observed in the absence or presence of rCyp19a1a (Supplementary Figure S1). In cultured ovarian tissues, the qPCR results showed that the expression of *cyp19a1a* was significantly higher in both low and high doses of rCyp19a1a than in the absence of rCyp19a1a (Figure 7A). In addition, *esr2a* expression was higher in both low- and high-dose Cyp19a1a over-expressed groups (Figure 7C), while *estrogen receptor 1* (*esr1*) was not significantly upregulated in these two doses in Cyp19a1a over-expressed groups (Figure 7B). However, rCyp19a1a treatment had no effect on the expression of *arα*, *folliculogenesis specific bHLH transcription factor* (*figla*), and *hydroxysteroid 17-beta dehydrogenase 1* (*hsd17β1*) (Figure 7D–F). For cultured testicular tissues, rCyp19a1a treatment had no effect on the expression of *cyp19a1a* and *esr1* (Figure 8A,B). However, *esr2a* and *arα* were significantly upregulated in the presence of the high-dose rCyp19a1a group (Figure 8C,D).

### 2.5. The Influence of Different Hormones on Gonadal Tissue Culture

**Figure 9:**
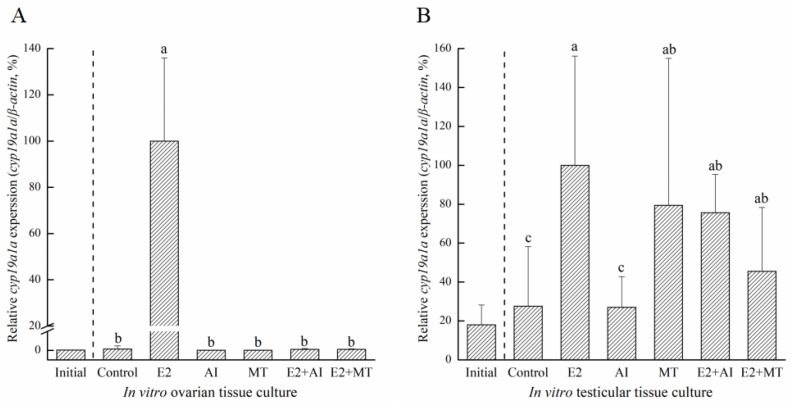
Effects of different hormone treatments on *cyp19a1a* expression in ovarian (**A**) and testicular (**B**) tissues. Initial, *cyp19a1a* expression before the treatment experiment. E2, 50 ng/mL of β-estradiol; MT, 120 ng/mL of methyltestosterone solution; AI, 1000 ng/mL of anastrozole; E2+AI, 50 ng/mL of β-estradiol and 1000 ng/mL of anastrozole; E2+MT, 50 ng/mL of β-estradiol and 120 ng/mL of methyltestosterone solution. Superscript letters indicate one-way ANOVA, followed by Duncan’s test (*p* < 0.05). Superscript letters indicate one-way ANOVA, followed by Tukey’s test (*p* < 0.05).

To analyze the proliferation activity of ovaries or testis in different hormones, IHC was used to detect the incorporation of BrdU. The BrdU-incorporated cells were observed in the different hormones treatment (Supplementary Figure S2). Furthermore, according to the results of BrdU IHC staining, the number of proliferated cells were counted (Supplementary Figure S3). According to the count, the different concentration groups with the highest cell proliferation count were selected as the optimal concentration. The 50 ng/mL E2 concentration was selected as the optimal concentration in the ovary (Supplementary Figure S2C). For the AI and MT concentrations, 1000 ng/mL and 120 ng/mL were selected, respectively (Supplementary Figure S2E,G). In testicular tissue culture, 100 ng/mL E2, 100 ng/mL AI, and 15 ng/mL MT concentrations had the highest numbers of proliferated cells (Supplementary Figure S2D,F,G). Therefore, the optimal concentrations of different hormones or medicine were selected to add to the culture medium. The results of different hormone treatments showed that *cyp19a1a* was expressed significantly in the E2 group than compared to the other groups in both ovarian tissue culture and testicular tissue culture (Figure 9). Also, in testicular tissue culture, excepted the E2 group, *cyp19a1a* expression in MT, E2+AI, and E2+MT groups was significantly higher than the control and AI groups (Figure 9B). Taken together, E2 may induce *cyp19a1a* expression in in vitro gonadal tissue culture.

## 3. Discussion

### 3.1. Duplicated Aromatase Genes in P. argenteus

The *cyp19a1* genes in teleosts are different from most other vertebrates because they have two forms, namely *cyp19a1a* and *cyp19a1b* [21]. Up to now, these two forms of aromatase gene have been identified in many teleosts, such as rainbow trout (*Oncorhynchus mykiss*) [22,23], zebrafish (*Danio rerio*) [24], Nile tilapia (*Oreochromis niloticus*) [25], European sea bass (*Dicentrarchus labrax*) [26], red spotted pufferfish (*Takifugu rubripes*) [20], orange-spotted grouper (*Epinephelus coioides*) [27], slender wrasse (*Halichoeres tenuispinis*) [28], and Asian swamp eel (*Monopterus albus*) [29]. The duplicated *cyp19a1* genes are assumed to be the result of the teleost-specific third round of whole-genome duplication (TWGD, 3R) [30]. Meanwhile, these two aromatase genes exhibit tissue-specific expression patterns, with *cyp19a1a* primarily expressed in the gonad and *cyp19a1b* predominantly expressed in the brain [31]. In this study, the *cyp19a1a* and *cyp19a1b* genes were identified in *P. argenteus*. According to the phylogenetic tree, Cyp19a1a and Cyp19a1b of *P. argenteus* clustered with their homologous genes in other fishes. However, although Cyp19a1a and Cyp19a1b were identified by the phylogenetic tree, the drawbacks of protein sequences, which were obtained from NCBI GenBank data, are that they may be influenced by errors in species identification. Also, GenBank records may lack detailed information regarding experimental methods, specimen sources, or other crucial experimental conditions. Protein sequences obtained from the NCBI database have certain limitations, such as some protein sequences were not identified as brain-type or ovarian-type aromatase. In this study, protein sequences annotated with ovarian aromatase and brain aromatase, or those with experimental results, were screened to minimize associated risks. Furthermore, *cyp19a1a* is mainly expressed in the gonad, whereas *cyp19a1b* is expressed in the brain, especially in the pituitary in this study. Therefore, the aromatase gene in *P. argenteus* was similar to that of most teleosts, exhibiting two forms which were brain type and ovarian type.

### 3.2. The Ovarian-Type Aromatase Might Play an Important Role in Ovarian Differentiation and Maturation

In teleosts, the gonadal aromatase gene *cyp19a1a* mainly expresses in the gonad and plays a role in early embryo development and gonadal sex differentiation, contributing to the initiation and maintenance of ovarian differentiation [9,32]. *cyp19a1a* also participates in gonadogenesis and gametogenesis in mature fish [33]. In *P. argenteus*, *cyp19a1a* was mainly expressed in the ovaries, which is similar to rainbow trout [23], Asian swamp eel [29], and *Schizothorax prenanti* [16]. In addition, a particularly high expression was detected at stage II and stage III during testicular development, which was consistent with the results in *Scatophagus argus* [34] and *Heteropneustes fossilis* [19]. In *P. argenteus*, high *cyp19a1a* expression in the ovary was observed in stage IV and stage V during gonadal development, which was in agreement with findings in rainbow trout [22], *Pagrus major* [35], Nile tilapia [36], Asian swamp eel [29], and *S. prenanti* [16], showing that *cyp19a1a* significantly increased at the stage of vitelline accumulation. Meanwhile, *cyp19a1a* barely expressed during the early developmental stages of *P. argenteus*. This result was different from *Colossoma macropomum* in which the expression of *cyp19a1a* increased when the body length of fish was 2 to 4 cm [37]. However, in *P. argenteus*, the expression of *cyp19a1a* was higher in early differentiated ovary than in undifferentiated gonad [38], which indicated that *cyp19a1a* may play a role in triggering the initiation of female sex differentiation. Also, *cyp19a1a* was closely associated with early gonadal differentiation in Nile tilapia [25,39], *Oryzias luzonensis* [40], and *Cyprinus carpio* [41]. Thus, the *cyp19a1a* of *P. argenteus* may play an important role in ovarian differentiation.

The regulation of ovarian maturation in fish by aromatase plays a crucial role in reproductive processes [42]. Aromatase is responsible for the conversion of androgens to estrogens, affecting various aspects of ovarian development, oocyte maturation, and the reproductive cycle. During the early stages of ovarian maturation, aromatase activity is relatively low, coinciding with elevated androgen levels [32]. As ovarian maturation progresses, there is a dynamic upregulation of aromatase expression, leading to an increased conversion of androgens to estrogens. This hormonal shift is essential for the development and maturation of oocytes within the ovaries [43]. In *P. argenteus*, *cyp19a1a* expression in ovaries was significantly higher than in testis, with the highest expression at stage V in ovaries during gonadal development, which indicated that the ovarian-type aromatase might play an important role in ovarian maturation in *P. argenteus.*

### 3.3. The Brain-Type Aromatase Might Be Involved in the Regulation of Both Brain and Gonadal Development

Aromatase activity in the brain is significantly more pronounced in teleosts compared to mammals. This difference has been proposed to contribute to the plasticity observed in the teleost brain [44,45]. The expression of *cyp19a1b* in teleost brain is related to brain cell proliferation and plays a crucial role in brain development, neurogenesis, and brain repair [46,47]. In the present study, *cyp19a1b* was expressed specifically in the brain, especially in the pituitary of *P. argenteus*. And during gonadal development, *cyp19a1b* was highly expressed at stage III to stage IV in the male pituitary, and stage IV to stage V in the female pituitary. This expression pattern in *P. argenteus* was similar to zebrafish [48], pufferfish [20], and the black porgy (*Acanthopagrus schlegelii*) [49]. In addition, it was found that the mutation of *cyp19a1b* increased the latency to the first oviposition in female zebrafish, indicating that *cyp19a1b* participates in the regulation of gonadal development. Taken together, *cyp19a1b* might be involved in brain development and also participate in the regulation of gonadal development in *P. argenteus*.

### 3.4. Cyp19a1a Participates in E2 Synthesis through Co-Regulation with esr2a

Aromatase is responsible for estrogen production, together with estrogens. It is generally considered to be essential for ovarian differentiation and plays a direct role in regulating ovary development and maturation [50,51]. In teleosts, such as Nile tilapia [52], zebrafish [53], and medaka (*Oryzias luzonensis*) [54], the knockout of *cyp19a1a* through genome editing methodologies leads to the development of testes. In numerous teleosts, genotypic males go into feminization after E2 treatment [55]. The methods of E2 or aromatase inhibitor treatment have been extensively employed for sex ratio control [56,57,58,59]. In this study, after the addition of different hormones, the expression of *cyp19a1a* was highest in the E2 addition group in the ovary and *cyp19a1a* expression was higher in E2 and MT groups in testes, which indicated that there existed a complex feedback regulation mechanism between aromatase and estrogen receptors. An increase in estrogen levels can stimulate the synthesis of aromatase. Also, in this study, *cyp19a1a* expression was significantly increased after rCyp19a1a treatment in both low and high doses in ovarian tissue, which indicated the effectiveness of Cyp19a1a in the ovary. *esr2a* was significantly increased after low-dose rCyp19a1a treatment. Although there were no significant differences in the femaleness-related genes, the expression of *esr1*, *figla*, and *hsd17β1* genes increased after rCyp19a1a overexpression. In testicular culture, the expression of *esr2a* and *ara* was significantly increased. These data indicated that Cyp19a1a in *P. argenteus* was responsible for androgens converting into estrogens, which was similar to most teleosts. These data suggested that estrogen receptors regulated the expression of the aromatase gene directly or indirectly. The binding of estrogen could influence the transcription levels of the aromatase gene, thereby regulating the synthesis of estrogen. Studies indicated that estrogen receptors were widely expressed in ovarian tissue, divided into two main subtypes, *esr1* and *esr2* [60]. The expression patterns of these two subtypes undergo dynamic changes during different developmental stages and reproductive cycles [61]. Aromatase, through regulating the expression and activity of these estrogen receptors, directly influences the activation of the estrogen signaling pathway [62]. Thus, Cyp19a1a is involved in the synthesis of E2 through co-regulation with *esr2a* in *P. argenteus*.

## 4. Materials and Methods

### 4.1. Animal and Sample Collection

*P. argenteus* were reared at Xiangshan Bay, Zhejiang, China. All fish experiments were conducted in accordance with the recommendations of the National Institutes of Health Guide for the Care and Use of Laboratory Animals. The Animal Care and Use Committee of Ningbo University approved the protocols (permit no. NBU20220079). We performed year-round sampling of cultured *P. argenteus*. The larvae of *P. argenteus* were collected from April 2022 to May 2022 and stored at −80 °C immediately. The gonads from stage I to stage VI of *P. argenteus* were sampled from July 2022 to August 2023. Monthly, random sampling was conducted with 20 fish. In total, 260 fish were collected. The body weight of sampled fish was from 4.1 g to 86.21 g, and the body length was from 5.7 cm to 18.2 cm. For sampling, MS222 (100 mg/L, Tricaine methanesulfonate, Sigma-Aldrich Co. LLC., St. Louis, MO, USA) was used to anesthetize the fish. One side of each gonad was used for histological observations to validate the gonadal status, while the other side of the gonad was used for qPCR experiments after gonadal status classification.

### 4.2. Gonadal Histology

The fish gonads were fixed with 4% paraformaldehyde in PBS at 4 °C for 12 h and then dehydrated using 100% methanol. After that, the gonads were embedded in paraffin wax and then cut into 5 μm thick sections. Finally, the gonadal sections were rehydrated in PBS and then stained with hematoxylin and eosin (H&E). The stages of gonadal development of all fish species in this study were examined histologically.

### 4.3. Total RNA Extraction and cDNA Synthesis

After the gonadal status of all fish had been determined via histology, six different stages of gonads were used to ascertain gene expression patterns. Extraction of total RNA followed the TransZol Up Kit (Transgene, ET111) manufacturer’s protocol. The quantity of total RNA was determined using a NanoDrop™ 1000 spectrometer (Thermo Fisher Scientific, Waltham, MA, USA). The first-strand cDNA was synthesized from 1 μg total RNA with HiFiScript gDNA Removal RT MasterMix Kit (CWBIO, Beijing, China). cDNA synthesis was performed using the manufacturer’s protocol.

### 4.4. Cloning of cyp19a1a and cyp19a1b cDNA

Full-length cDNA sequences of the *P. argenteus cyp19a1a* and *cyp19a1b* genes were amplified using PCR. Degenerate primers were designed based on conserved regions of the gene from other fish species. The primers used for amplification are listed in Table 1.

### 4.5. Sequence Alignment and Phylogenetic Analysis

For phylogenic analysis, a set of Cyap19a1a and Cyp19a1b sequences in different fish species were retrieved from GenBank. The deduced amino acid sequences of *P. argenteus* Cyp19a1a and Cyp19a1b were subjected to multiple sequence alignment using Clustal W. The phylogenetic tree was constructed utilizing the Neighbor-Joining method within Molecular Evolutionary Genetics Analysis (MEGA) software version 7.0. The evolutionary distances were computed using the Jones–Taylor–Thornton (JTT) matrix-based method and are in the units of the number of amino acid substitutions per site. The rate variation among sites was modeled with a gamma distribution (shape parameter = 0.7). The number displayed at each node signifies the bootstrap probability (expressed as a percentage from 1000 replicates). Branches corresponding to partitions reproduced in less than 30% bootstrap replicates are collapsed. The accession numbers for the sequences obtained in the analysis can be found in the legend of Figure 1.

### 4.6. Quantitative Real-Time PCR

Gene quantification of standards, samples, and controls were conducted simultaneously using quantitative real-time PCR (qPCR) (Quantagene q225 Real-Time PCR System) with the MagicSYBR mixture (CWBIO, Beijing, China). *beta-actin* (*β-actin*; GenBank accession no. KF982333) was used as an internal control to normalize the gene expression level. Specific primers for *cyp19a1a, cyp19a1b, esr1*, *esr2a*, *arα*, *figla*, and *hsd17β1* are listed in Table 1. The specificity of PCR was confirmed through a single melting curve of unknown samples and standards. No signal was detected in non-template controls via qPCR. The data were calibrated according to the 2^−ΔΔCt^ method. The reaction efficiency of different genes was assessed through the analysis of serially diluted cDNA templates. Based on linear regression analysis, the accuracy of real-time PCR assays was high (R^2^ > 0.99) across all dilutions. The relative expression values of target genes in all samples were normalized to *β-actin*, and the highest value of the target genes was set as 100%.

### 4.7. Recombinant Cyp19a1a Protein Production

The open reading frame (ORF) of *cyp19a1a* was introduced into the pcDNA 3.1 (+) Mammalian Expression Vector (Thermo Fisher Scientific, Waltham, MA, USA). The PCR primers 5′-CTAGCTAGCATGACTCCTGTCGGTTTGGACAC-3′ and 5′-CCCAAGCTTTTACCAGCTGCCTCTCTTTCTTG-3′ were used to amplify the ORF of *cyp19a1a*. The 293T (human embryonic kidney) cell line was used as a host for the recombinant construct. The recombinant Cyp19a1a (rCyp19a1a) was produced in 293T cells as in previous studies [21,63], and was used to check the specificity of the anti-Cyp19a1a antibody via Western blot analysis. Also, rCyp19a1a was used to elucidate the function of Cyp19a1a through the in vitro testicular and ovarian tissue culture experiments.

### 4.8. Western Blot Analysis

The rat polyclonal antibody was generated against the full-length amino acid of *P. argenteus* Cyp19a1a. The antisera were prepared by Ai Ting Biotechnology, Inc., Hangzhou, China. The specificity of anti-Cyp19a1a antiserum was confirmed via Western blotting analysis and used for immunohistochemical (IHC) staining in this study. Gonadal protein was extracted with RIPA buffer (R0010, Solarbio, Beijing, China). β-Actin was detected using β-Actin Rabbit mAb (AC026, ABclonal, Wuhan, China) (diluted 1:5000 with 1.5% nonfat milk powder) and alkaline phosphatase-conjugated Affinipure Goat Anti-Rabbit IgG (H+L) (SA00002-2, Proteintech, Wuhan, China) (diluted 1:2000 with 1.5% nonfat milk powder), which functioned as primary antibody and secondary antibody. Anti-Cyp19a1a antiserum, which was diluted 1:1000 with 1.5% nonfat milk powder, was used to detect rCyp19a1a and Cyp19a1a. Alkaline phosphatase-conjugated affinipure goat anti-rat IgG (H+L) antibody (SA00002-9, Proteintech, Wuhan, China), which was diluted 1:2000 with 1.5% nonfat milk powder, was used for the secondary reaction. After that, the NBT/BCIP Detection System (PR1100, Solarbio, Beijing, China) was used to visualize the immunoreactivity.

### 4.9. IHC Analyses

For IHC staining, we followed the methodology described previously [63]. The gonadal sections were cut into 5 μm thick sections, and the rehydrated sections were treated with sodium citrate antigen retrieval buffer (PR30001, Proteintech, Wuhan, China) to expose the antigens of the target protein. Primary antibodies against Cyp19a1a, which were diluted 1:100 with nonfat milk, were used to detect Cyp19a1a. For the secondary antibody reactions, biotin-SP-conjugated affinipure goat anti-rat IgG (H+L) antibody (SA00004-8, Proteintech, Wuhan, China diluted 1:200) was used to detect the Cyp19a1a antibody. Also, anti-bromodeoxyuridine (BrdU) polyclonal antibody (K010024P, ABclonal, Wuhan, China) (diluted 1:100 with nonfat milk) and HRP goat anti-rabbit IgG (H+L) (AS014, ABclonal, Wuhan, China) (diluted 1:200 with nonfat milk) were used to detect cell proliferation. Immunoreactivity was amplified with an SABC-HRP kit (P0603, Beyotime, Nanjing, China). After that, 3, 3′-diaminobenzidine (DAB, P0202, Beyotime, Nanjing, China) was used to visualize the immunoreactivity.

### 4.10. rCyp19a1a Treatment

Gonadal tissue culture followed previous studies [21,63]. In brief, the ovary or testis was dissected into less than 1 mm thick pieces. The gonadal tissues, which involved six replicates in each group, were incubated in the presence or absence of rCyp19a1a for 7 days at 26 °C. The basal culture medium consisted of Leibovitz’s L-15 Medium (11415064, Gibco, Thermo Fisher Scientific, Waltham, MA, USA) supplemented with 10% fetal bovine serum and 2% penicillin–streptomycin liquid (P1400, Solarbio, Beijing, China). The low dose of rCyp19a1a was 15 μg/mL and the high dose of rCyp19a1a was 90 μg/mL. BrdU was used to analyze the effects of rCyp19a1a on the proliferation of mitotically active germ cells. Gonadal tissues were collected after 48 h of BrdU (60 μg/mL) treatment for immunohistology and gene expression analysis.

### 4.11. Sex Hormone Treatment

For sex hormone treatments, different concentrations of sex hormones including β-estradiol (GC11282, GLPBIO, Montclair, CA, USA) (E2, 0, 25, 50, 100, 200 ng/mL), methyltestosterone solution (GC19961, GLPBIO, Montclair, CA, USA) (MT, 0,15, 30, 60, 120 ng/mL), and anastrozole (GC10256, GLPBIO, Montclair, CA, USA) (aromatase inhibitor, AI, 0, 100, 1000, 10,000, 50,000 ng/mL) were used to culture gonadal tissue. BrdU was used to analyze the cell proliferation for each group. BrdU-incorporated cells were determined via IHC with anti-BrdU antibody. Sections of embedded gonadal tissues were collected from anterior, middle, and posterior parts for IHC staining. Sections were used to count the BrdU-incorporated cells. After counting, the best concentration of each sex hormone treatment was used for gene expression analysis.

### 4.12. Data Analysis

The data are presented as the mean ± SD (standard deviation). The Shapiro–Wilk method was used to analyze the normality of data distribution. The values were subjected to analysis via one-way ANOVA, followed by Tukey’s test or Duncan’s test, with *p* < 0.05 indicating a significant difference.

## 5. Conclusions

In this study, we demonstrated that the aromatase gene in *P. argenteus* exhibits two forms, which are brain-type aromatase and ovarian-type aromatase. *cyp19a1b* might be involved in brain development and also participates in the regulation of gonadal development, and *cyp19a1a* may play an important role in ovarian differentiation and ovarian maturation. Cyp19a1a is also involved in the synthesis of E2 through co-regulation with *esr2a* in *P. argenteus*.

## Figures and Tables

**Table 1:** Oligonucleotides for specific primers.

Primer Name	Gene	Oritetation	Length (bp)	Sequence
Clone	*cyp19a1a*	Sense	1591	5′-GATTTGATCTTGGCTTGTGAGCAGG-3′
		Antisense		5′-CACCACATAATGTTTGCACAGCCAT-3′
	*cyp19a1b*	Sense	1763	5′-ATGGTCTGATGACAAACTAACA-3′
		Antisense		5′-TCCTAAATACAACGGTGCTT-3′
Specific qPCR	*β-actin*	Sense	212	5′-ACCCAGATCATGTTCGAGACC-3′
		Antisense		5′-ATGAGGTAGTCTGTGAGGTCG-3′
	*cyp19a1a*	Sense	155	5′-CTCTCTCCATCAGCCTCTTCTTC-3′
		Antisense		5′-GTTGATGAAGCTCTCCAGCACCTG-3′
	*cyp19a1b*	Sense	210	5′-GCCCAAGAGCTACAAGATGTAATG-3′
		Antisense		5′-GAAGAAGAGGCTGATGGAAAGTG-3′
	*esr1*	Sense	115	5′-CTCCACCACTGGCTACTACTCTACTCC-3′
		Antisense		5′-GCTGGAGGGCACAAACACAAGAG-3′
	*esr2a*	Sense	165	5′-CCACTATCTTCAGCTATGCTGGCC-3′
		Antisense		5′-ACGGACTCTGCATTGGTTGGC-3′
	*arα*	Sense	139	5′-CAACAGTTCTGCATGCTGAACGTCAC-3′
		Antisense		5′-GCTCCTTTATGTAGGAGGTCCGCAG-3′
	*figla*	Sense	148	5′-TTGAAGCGGCTGACTGGCGA-3′
		Antisense		5′-TCAGCCGTTCCTTGGCGTTG-3′
	*hsd17β1*	Sense	204	5′-CAGCTGGATACACACACACTCAGC-3′
		Antisense		5′-CAAGCCATCTGGTTCAGTGAGCTTC-3′

## Data Availability

Data is contained within the article and Supplementary Material.

## Supplementary Materials

The following supporting information can be downloaded at: https://www.mdpi.com/article/10.3390/ijms25031583/s1.
