# Supplementary material for: Cyp19a1a Promotes Ovarian Maturation through Regulating E2 Synthesis with Estrogen Receptor 2a in Pampus argenteus (Euphrasen, 1788)
Source: Int J Mol Sci. 2024 Jan 27;25(3):1583. doi: 10.3390/ijms25031583 (PMC10855460; doi:10.3390/ijms25031583)
Supplement: Supplementary file 1 [file ijms-25-01583-s001.zip › Supplemental Figure S1-3 Legends.pdf]

Supplemental Figure S1. IHC staining of BrdU to detect the number of cell proliferation in low or high dose of rCyp19a1a treatment. The proliferation activity of the rCyp19a1a or control group. The low dose of rCyp19a1a treatment (A) and its control group (B) in ovary. The high dose of rCyp19a1a treatment (C) and its control group (D) in ovarian tissue. The low dose of rCyp19a1a treatment (E) and its control group (F) in testicular tissue. The high dose of rCyp19a1a treatment (G) and its control group (H) in testicular tissue. The black arrowheads indicate the signals of the anti-BrdU antibody.

Supplemental Figure S2. IHC staining of BrdU to detect the number of cell proliferation in E2, AI, and MT treatment. The proliferation activity of the control group in female fish (A) and male fish (B), respectively; E2 treatment in female fish (C) and male fish (D), respectively; AI treatment in female fish (E) and male fish (F), respectively; MT treatment in female fish (G) and male fish (H), respectively. The black arrowheads indicate the signals of the anti-BrdU antibody.

Supplemental Figure S3. Effects of E2, MT, and AI on the number of cell proliferation. The proliferateion activity of the germline cells were counted by the number of BrdU-incorporated cell. The proliferation activity of the (A) β-estradiol, (B) methyltestosterone, and (C) anastrozole treatment in ovarian tissue (n = 6). The proliferation activity of the (D) β-estradiol, (E) methyltestosterone, and (F) anastrozole treatment in testicular tissue (n = 6). Superscript letters indicate one-way ANOVA, followed by Duncan's test ($P < 0.05$).
