# Supplementary figures and images for: Cyp19a1a Promotes Ovarian Maturation through Regulating E2 Synthesis with Estrogen Receptor 2a in Pampus argenteus (Euphrasen, 1788)
Source: Int J Mol Sci. 2024 Jan 27;25(3):1583. doi: 10.3390/ijms25031583 (PMC10855460; doi:10.3390/ijms25031583)

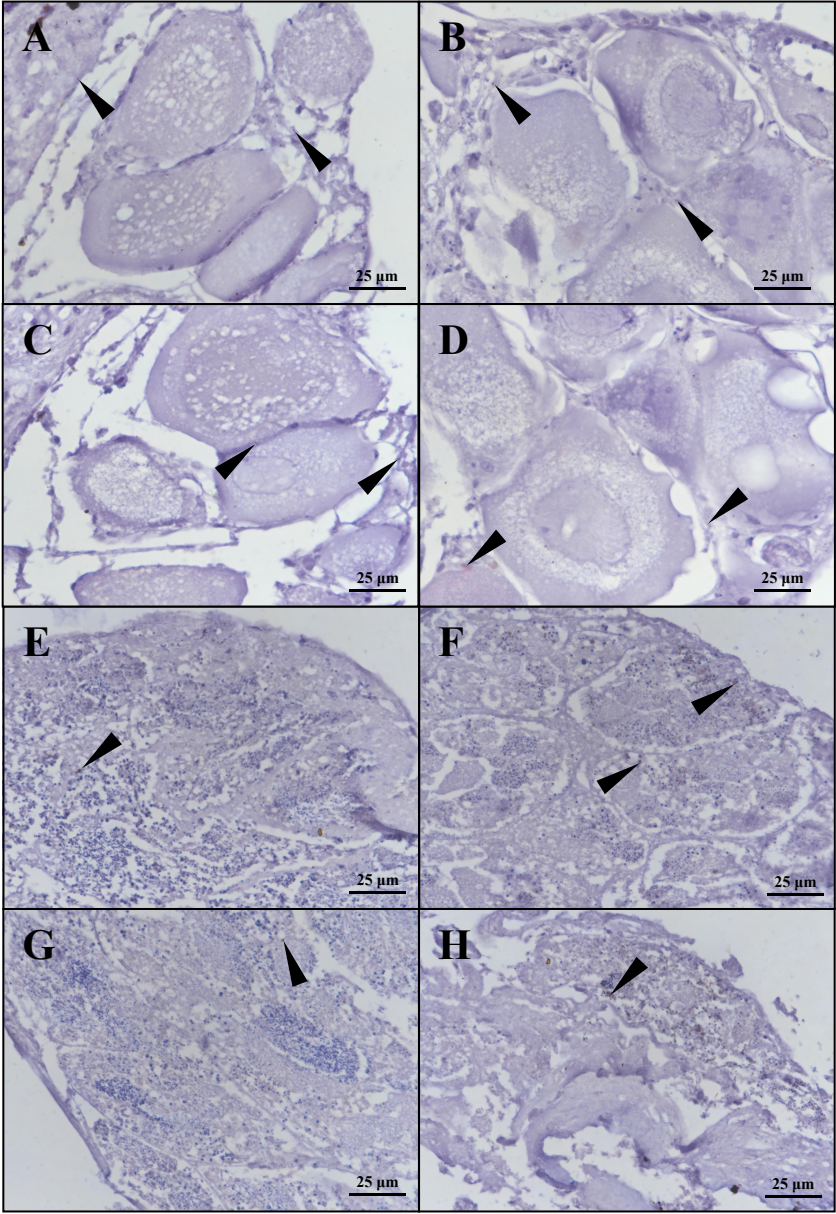

Supplementary Figure S1

Supplement: Supplementary file 1 [file ijms-25-01583-s001.zip › Supplementary Figure S1.pdf]

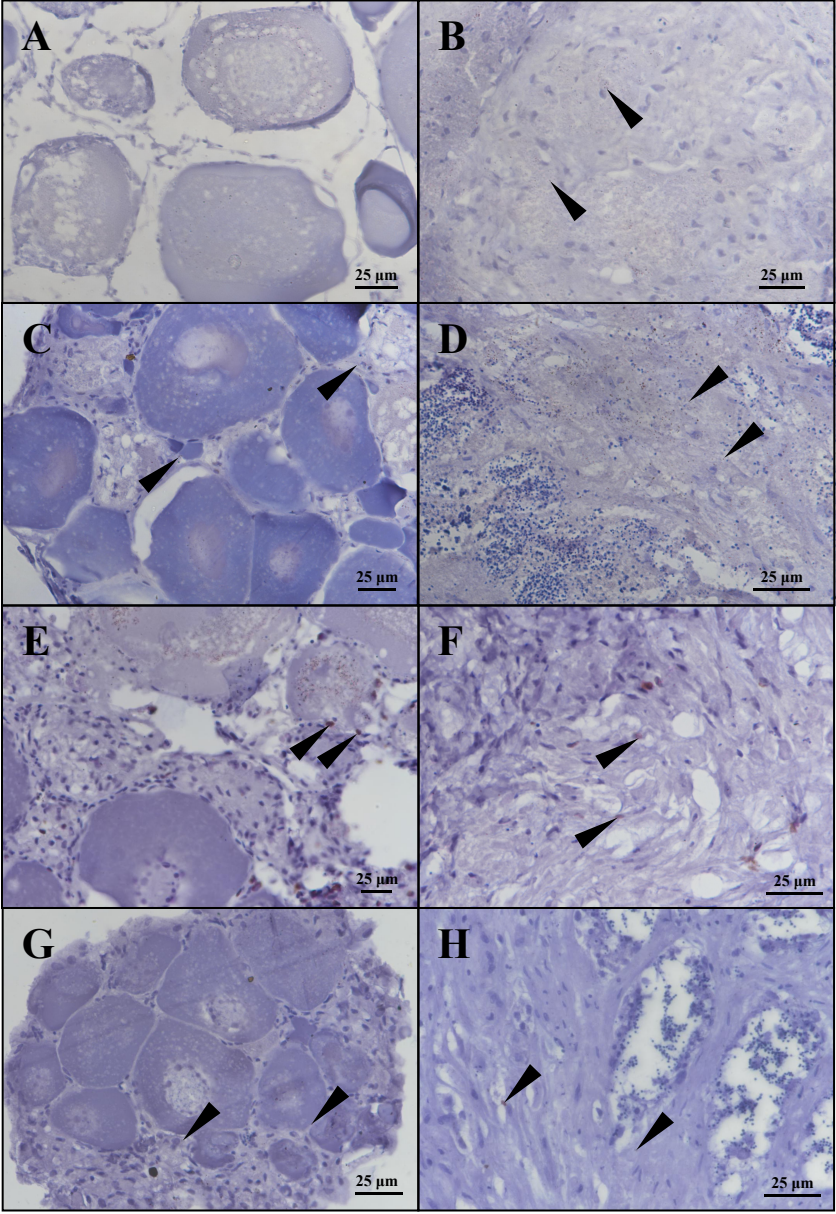

Supplementary Figure S2

Supplement: Supplementary file 1 [file ijms-25-01583-s001.zip › Supplementary Figure S2.pdf]

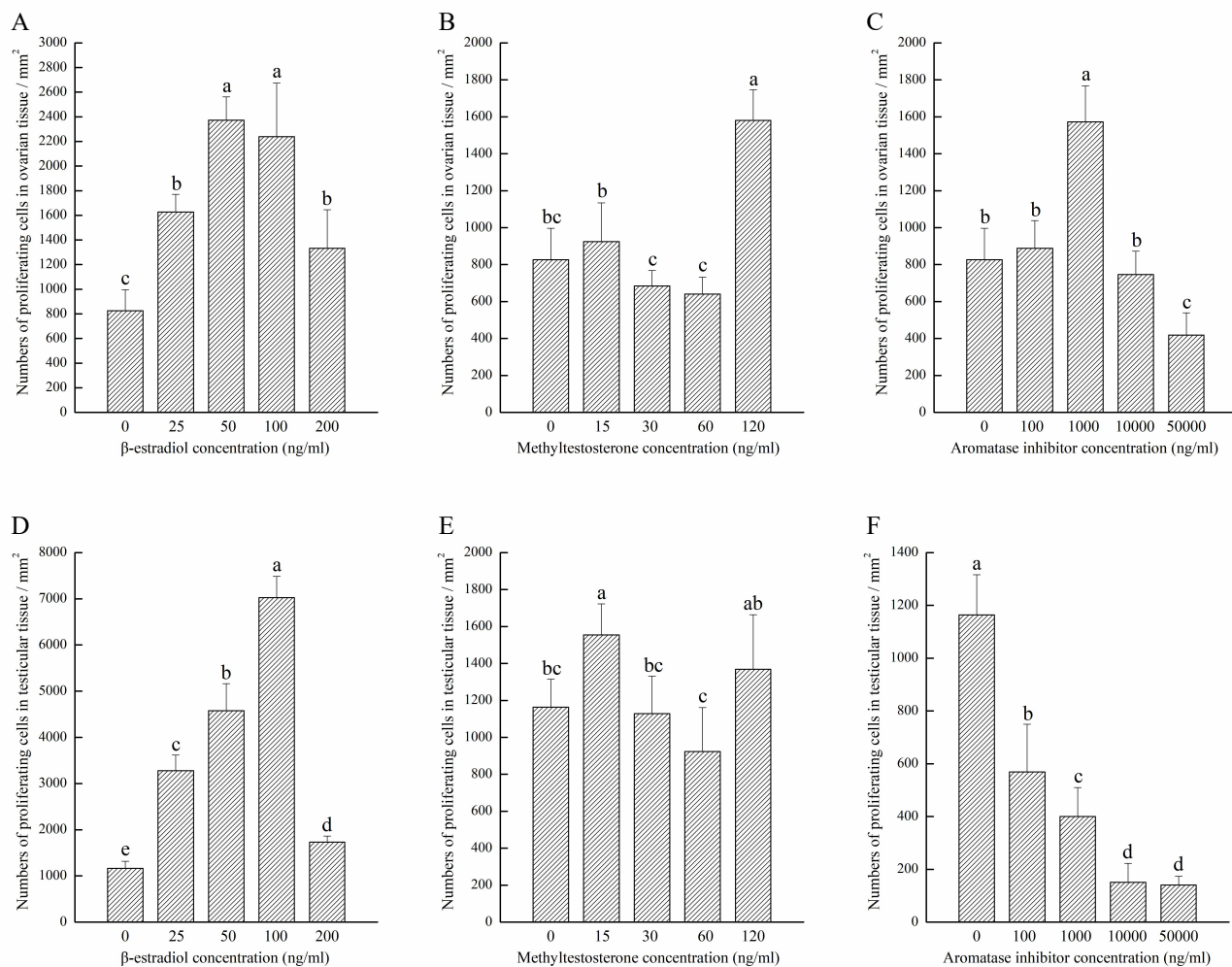

Supplementary Figure S3

Supplement: Supplementary file 1 [file ijms-25-01583-s001.zip › Supplementary Figure S3.pdf]
